# The Spanish Adaptation of the Quality in Psychiatric Care—Forensic Inpatient (QPC-FIP) Instrument: Psychometric Properties

**DOI:** 10.3390/healthcare12222235

**Published:** 2024-11-09

**Authors:** Marta Domínguez del Campo, Juan Roldán-Merino, Manuel Tomás-Jiménez, Montserrat Puig-Llobet, Maria Teresa Lluch-Canut, Nathalia Rodríguez Zunino, Sara Sanchez-Balcells, Agneta Schröder, Lars-Olov Lundqvist, Gemma Escuder-Romeva, Antonio R. Moreno-Poyato

**Affiliations:** 1 Parc Sanitari Sant Joan de Déu, Dr. Antoni Pujades 42, 08830 Sant Boi de Llobregat, Spain; marta.dominguez@sjd.es (M.D.d.C.); nathalia.rodriguez@sjd.es (N.R.Z.); gemma.escuder@sjd.es (G.E.-R.); 2 Etiopatogenia I Tractament Dels Trastorns Mental Severs (MERITT), Institut de Recerca Sant Joan de Déu, Santa Rosa 39-57, 08950 Esplugues de Llobregat, Spain; 3 Fundació Privada Per La Recerca Sant Joan de Déu, Santa Rosa 39-57, 08950 Esplugues del Llobregat, Spain; 4 Mental Health Department, Campus Docent Sant Joan de Déu Private Foundation, University of Barcelona, C/Sant Benito Menni 18-20, 08830 Sant Boi de Llobregat, Spain; juan.roldan@sjd.edu.es; 5 Grupo DAFNiS, Campus Docent Sant Joan de Déu, Universitat de Barcelona, 08830 Sant Boi de Llobregat, Spain; 6 Mental Health, Psychosocial and Complex Nursing Care Research Group—NURSEARCH, University of Barcelona, 08907 Barcelona, Spain; monpuigllob@ub.edu (M.P.-L.); tlluch@ub.edu (M.T.L.-C.); sara.sanchez@ub.edu (S.S.-B.); amorenopoyato@ub.edu (A.R.M.-P.); 7 Patient Safety Research Group, Parc Sanitari Sant Joan de Déu, 08830 Sant Boi de Llobregat, Spain; 8 Public Health, Mental Health and Maternal Infant Nursing Department, Nursing College, University of Barcelona, Health Sciences Campus Bellvitge, Hospitalet de Llobregat, 08907 Barcelona, Spain; 9 University Health Care Research Center, Faculty of Medicine and Health, Örebro University, 701 85 Örebro, Sweden; agneta.schroder@regionorebrolan.se (A.S.); lars-olov.lundqvist@regionorebrolan.se (L.-O.L.); 10 Department of Nursing, Faculty of Health Care and Nursing, Norwegian University of Science and Technology (NTNU), 2815 Gjövik, Norway

**Keywords:** forensic care, psychometric properties, patients’ perspective, quality of care, psychiatric care

## Abstract

**Background/Objectives**: The quality of care in forensic mental health services is a factor that significantly impacts recovery and constitutes a right of the individuals receiving treatment. However, there is a lack of instruments to assess the perceived quality of care among individuals in this setting. Quality in Psychiatric Care—Forensic Inpatient (QPC-FIP) is a Swedish instrument that measures the perception of quality care from the perspectives of patients in the forensic setting. The aim of this study was to perform a cross-cultural adaptation of the QPC-FIP instrument into Spanish and to assess its reliability and validity. **Methods**: For the adaptation process, a translation–backtranslation of the instrument was performed. Regarding psychometric properties, the sample consisted of 120 inpatients in the forensic setting to whom the instrument was applied. To assess temporal stability, the instrument was readministered after 10 days (*n* = 98). **Results**: The confirmatory factor analysis showed an equivalent seven-factor structure with the original version, presenting a satisfactory model fit. Regarding reliability, the Cronbach’s alpha value was 0.933, and the intraclass correlation coefficient was 0.836 (95% IC: 0.742–0.896), revealing results higher than 0.70 in six of the seven factors. **Conclusions**: The Spanish version of the QPC-FIP instrument showed adequate validity and reliability values, indicating that is a useful tool for measuring quality in psychiatric care in the forensic context.

## 1. Introduction

The prison environment can be a predisposing factor that requires people to continually adapt to their changing circumstances. Incarceration, as a significant life event, creates a biographical disruption and can lead to changes in self-care habits and social networks for people involved in the justice system. However, it is important to recognize that mental health problems are not the exclusive consequence of incarceration. Factors such as previous trauma, limited access to health services and socioeconomic conditions also play a fundamental role in the development of these disorders. These elements interact with the characteristics of the prison environment, alterations in care routines and, in particular, deprivation of freedom, contributing to the emergence of psychological difficulties [1,2]. This approach facilitates a deeper understanding of the complexity of the lives of individuals involved in the justice system, highlighting the importance of support and treatment approaches that recognize their multifaceted experiences and respect their humanity.

Various studies indicate that, at present, the prison system is the institution housing the majority of individuals with mental health problems [3,4,5,6]. In Spain, the prevalence of mental health issues within forensic settings is 84.4% [7,8,9]. In relation to mental health within the Catalonian prison system, a 2018 study aimed at determining the needs of the population served by the Psychiatric Hospitalization Unit of Catalonia (UHPP), where a large portion of the present study’s sample is located, found that 70% of the population had a diagnosis of “Schizophrenia spectrum and other psychotic disorders”, followed by 23% of individuals diagnosed with “Mood disorders” [10].

The Organic Law 1/1979, of September 26, General Penitentiary Law, establishes the minimum standards that penitentiary institutions in Spain must meet to ensure the fulfillment of the basic needs of the inmate population. This includes, as outlined in Articles 19 to 21, maintaining personal and environmental hygiene, providing adequate nutrition, and ensuring the right to social activity and relationships, among other aspects. Additionally, Article 61 emphasizes the promotion of inmate participation in the planning and execution of their treatment and discharge planning [11].

The quality of care in forensic mental health services is a factor that significantly impacts the recovery and quality of life of the individuals receiving treatment [12]. The right to high-quality mental health care is a fundamental principle supported by international organizations such as the World Health Organization (WHO) and the Convention on the Rights of Persons with Disabilities (CRPD). However, global evidence indicates that mental health services, particularly in forensic settings, often face significant shortcomings [13].

Referring to the recovery framework in mental health, the importance of promoting autonomy, choice, and participation in decision making is emphasized in forensic environments. These aspects are often compromised in forensic settings due to the strict security and control measures in place [12,14].

Furthermore, the literature highlights that environments promoting recovery and maintaining a high quality of life are those characterized by high-quality therapeutic relationships, a welcoming physical environment, and opportunities to engage in meaningful activities that support care and recovery [15]. These findings are consistent with studies that have examined the quality of life of forensic psychiatric inpatients, emphasizing that improvements in the quality of care can have a significant impact on the recovery and reintegration of inmates, thereby reducing recidivism and improving health outcomes [2,7,9].

In the European context, forensic mental health remains a critical area of concern, with an emphasis on the need for greater integration of mental health services within the prison system. Differences in care models among European countries, along with variations in public health and penitentiary policies, pose challenges to the implementation of a uniform approach to address this issue [5].

In Catalonia, where the present study is conducted, the regional government has implemented a rehabilitation model in prisons aimed at improving the quality of life of inmates through the promotion of personalized treatment programs [16]. However, there is a lack of instruments to assess the perceived quality of care among individuals in forensic mental health units, a key element emphasized for the past two decades [17,18,19].

Furthermore, there is a lack of cross-cultural comparative studies on the perceptions of patients and professionals regarding the quality of care [20] particularly in forensic settings. This is primarily due to the absence of standardized instruments.

Regarding studies that assess the perception of quality of care in patients, it is important to highlight the work of Schröder and Ahlström and their development of the Quality in Psychiatric Care (QPC) instrument battery, which evaluates different dimensions characterizing the quality of mental health care across three distinct settings: hospital, community, and forensic. Each area has developed a specific self-administered instrument to assess the quality of care from the perspective of both professionals and patients [21]. In Spain, the respective instruments have been validated for mental health professionals in hospital settings [22], inpatients in psychiatric units [23], mental health professionals in community settings [24], and outpatients in community mental health centers [25].

The present research group has carried out various studies on quality of care in the context of mental health in Catalonia (Spain). The first area to explore was the hospital context, where it was observed that professionals gave a higher score to the dimensions referring to Support received as well as Encounter, a dimension that reflects the therapeutic relationship between professionals and patients [22]. Subsequently, the point of view of patients admitted to hospital units was examined, with patients agreeing with the professionals in giving the highest score to the dimensions Support and Encounter [23]. The next context to study was that related to community mental health, so perspectives on quality of care in professionals and patients were examined, and the results showed an agreement in giving higher scores to the dimensions related to Support and Encounter in a similar way to what was found in the hospital area [26].

Within the scope of our study, the QPC-FIPS (Quality in Psychiatric Care—Forensic Inpatient Staff) instrument, the QPC version for professionals in the field of prison mental health, has already been cross-culturally adapted to our context [27]. However, the QPC-FIP (Quality of Psychiatric Care—Forensic Inpatient) instrument [28], the QPC version for individuals receiving care in forensic mental health units, has not yet been validated in Spanish.

This study is part of an international project aimed at adapting the patient version of the QPC-FIP instrument across several countries, evaluating its psychometric properties and comparing how its structure holds across different languages. Additionally, the project aims to analyze and contrast the quality of psychiatric care in forensic settings across multiple nations.

In this context, the aim of this study was to perform a cross-cultural adaptation of the Quality in Psychiatric Care—Forensic Inpatient (QPC-FIP) instrument into Spanish and to assess its reliability and validity.

## 2. Materials and Methods

### 2.1. Design

To address the validation of the Spanish version of the QPC-FIP instrument, a psychometric study was conducted in two phases.

- Phase 1. Adaptation of the instrument through translation, back-translation, and pilot testing of cognitive pretest.
- Phase 2. Validation of the psychometric properties of the instrument through a study of the validity and reliability of the Spanish version.

### 2.2. Participants and Study Setting (Sample Size)

The study was conducted between January and December 2023. The sample consisted of inpatients admitted to the Forensic Psychiatry Hospitalization Units of Parc Sanitari Sant Joan de Déu: UHPP-C (Penitentiary Psychiatric Hospitalization Unit of Catalonia); Brians 2 Mental Health Unit, and Quatre Camins.

At the study site, the maximum capacity is approximately 120 beds (excluding beds reserved for emergencies and individuals with acute illness). It is worth noting that there is a low replacement rate because discharges from these units typically coincide with medical discharges or completion of the sentence. Due to this limitation, a non-probabilistic convenience sampling method was employed. To reduce sample bias, the data were collected by professionals outside the research team. It was agreed to include all individuals admitted during the study period who met the following inclusion criteria: having a diagnosis of mental disorder and agreeing to participate voluntarily in the research. Exclusion criteria were language barrier, significant cognitive impairment, organic disorder, and drug intoxication at the time of evaluation.

It was decided to include a minimum of 100 participants to assess internal consistency and to conduct confirmatory factor analysis, due to the unit’s low replacement rate [29]. To evaluate consistency over time, it was calculated that at least 80 participants would be needed to detect an intraclass correlation coefficient (ICC) close to 0.70 between two measurements, maintaining a 95% confidence level and 80% power for a two-sided comparison [30].

The validation of the psychometric properties of the instrument was conducted with the participation of 120 patients. Sociodemographic and clinical characteristics are shown in Table 1.

### 2.3. Variables and Sources of Information

The QPC-FIP instrument is a valid and reliable instrument for measuring the quality of care from mental health patient’s point of view in the forensic environment. Regarding reliability, this instrument showed satisfactory results (global Cronbach’s α = 0.98) [28].

The instrument consists of a total of 34 items distributed across 7 factors. Below are the specific characteristics of each factor, the number of items composing it, and the internal consistency of each factor in the original version:

- Factor 1. Encounter (8 items): Represents the therapeutic relationship between the patient and the professional, where the patient evaluates the level of empathy, respect, concern, and listening demonstrated by the professional. (Cronbach’s α in the original version = 0.96.)
- Factor 2. Participation (8 items): Refers to the patient’s perception of their involvement in care planning, informed treatment decision making, and their overall experience. (Cronbach’s α in the original version = 0.93.)
- Factor 3. Discharge (3 items): Evaluates the patient’s thoughts on the planning of post-discharge follow-up. (Cronbach’s α in the original version = 0.81.)
- Factor 4. Support (4 items): Represents the patient’s perception of support from professionals when experiencing self-harm, harm to others, or self-stigma. (Cronbach’s α in the original version = 0.86.)
- Factor 5. Secluded Environment (2 items): Refers to the availability of a private place for the patient. (Cronbach’s α in the original version = 0.62.)
- Factor 6. Secure Environment (3 items): Identifies the perception of safety within the forensic psychiatry unit. (Cronbach’s α in the original version = 0.72.)
- Factor 7. Forensic-Specific (6 items): Evaluates various elements related to the health care and judicial systems. (Cronbach’s α in the original version = 0.87.)

Each item begins with the words “I consider that…” and is scored on a Likert-type scale with 4 response options ranging from 1 (completely disagree) to 4 (completely agree).

Scores for assessing quality perceptions can be calculated either as total or by dimensions, having a total range of scores from 34 to 136. High scores both in the total instrument and each dimension can be interpreted as a positive perception of quality of care by mental health patients. Conversely, low scores indicate areas for improvement that need to be addressed to improve the quality of care.

Sociodemographic variables of the participants were registered, such as age, length of stay in the unit, type of treatment received, identification of referring professionals, belief in participation in the planning of their medical care, and perception of the level of health, among others.

### 2.4. Procedure

Phase I. For cross-cultural adaptation, the QPC-FIP scale has been translated into Spanish.

The translation and back-translation process was conducted following the Standards for Educational and Psychological Testing.

Firstly, the authors of the original scales granted permission for cross-cultural validation, and a collaboration agreement was established. Subsequently, the original version was translated into Spanish, based on the English version provided by the original author, by two independent native translators with no knowledge of the instruments or the study’s objectives.

Later, a panel of experts, including a psychometrics specialist, three quality experts, five mental health nurses, one psychologist, and one forensic medicine specialized psychiatrist, assessed the conceptual equivalence, semantic equivalence (synonymous words, challenges during translation) and idiomatic equivalence (informal expressions, contextualization of the text. The expert committee suggested modifying two elements to improve comprehension in their cross-cultural adaptation.

Finally, the Spanish versions of the instruments were obtained, which were then back translated into English and compared again with the original. Subsequently, a pilot study with cognitive pretesting was conducted with a sample of 30 patients in forensic psychiatry units to assess comprehension and completion time. The Spanish version of the QPC-FIP scale was ultimately developed.

Phase II. The second phase involves a psychometric study of the validity and reliability of the Spanish version of the instrument.

The principal investigator informed the study sample about the objectives and methodology of the study and personally attended the recruitment and data extraction from the study participants. After 10 days, a re-evaluation of the instrument was conducted to assess temporal stability. This interval of days was chosen taking in account the effect of memory as well as the possibility that significant changes in the participant’s mental state or relating to the environment could occur. Therefore, an interval of 10 days was considered prudent to capture stability in their perceptions of quality in psychiatric care. The re-evaluation was performed under similar conditions to ensure that participants were in a comparable state to the first evaluation.

### 2.5. Statistical Analysis

For the analyses, version 26 of the SPSS Statistics program, along with version 6.3 of the EQS program for confirmatory factor analysis (CFA), was used.

For the measurement of psychometric properties, factor validity and reliability were assessed.

- Factor validity. Confirmatory factor analysis was performed (CFA), with parameters estimated using the least squares method applying a polychoric correlation matrix. This procedure it is usually applied to ordinal items, and has comparable properties with the maximum likelihood method, but the criteria are less rigorous than those in typical procedures [31].

The following fit indices were calculated to evaluate the model’s overall fit:

Goodness-of-fit index (GFI), adjusted goodness-of-fit index (AGFI), comparative fit index (CFI), Bentler–Bonnet Normed Fit Index (BBNFI), Bentler–Bonnet Non-Normed Fit Index (BBNNFI), root mean square residual (RMR), standardized root mean square (SRMR), root mean square error of approximation (RMSEA), the chi-squared goodness-of-fit test, and the chi-squared-to-degree-of-freedom ratio (χ2/df). A good model fit was defined by an X^2^/df ratio < 3, GFI, AGFI, BBNFI, BBNNFI, and CFI values close to 0.90 [32,33,34] and RMR, SRMR, and RMSEA values below 0.08 [35].

- Reliability. Cronbach’s alpha and McDonald’s omega were calculated as indicators of internal consistency, considering values equal to 0.70 or greater as satisfactory [36]. This values were calculated for the total instrument and for each dimension.
- The corrected item-total correlation was analyzed, where correlations between each item with the global instrument and with each dimension were estimated. A lower limit of 0.20 was accepted. Temporal stability (test–retest stability) was assessed taking values equal to 0.70 or greater as an indicator of good agreement.

## 3. Results

**Figure 1:**
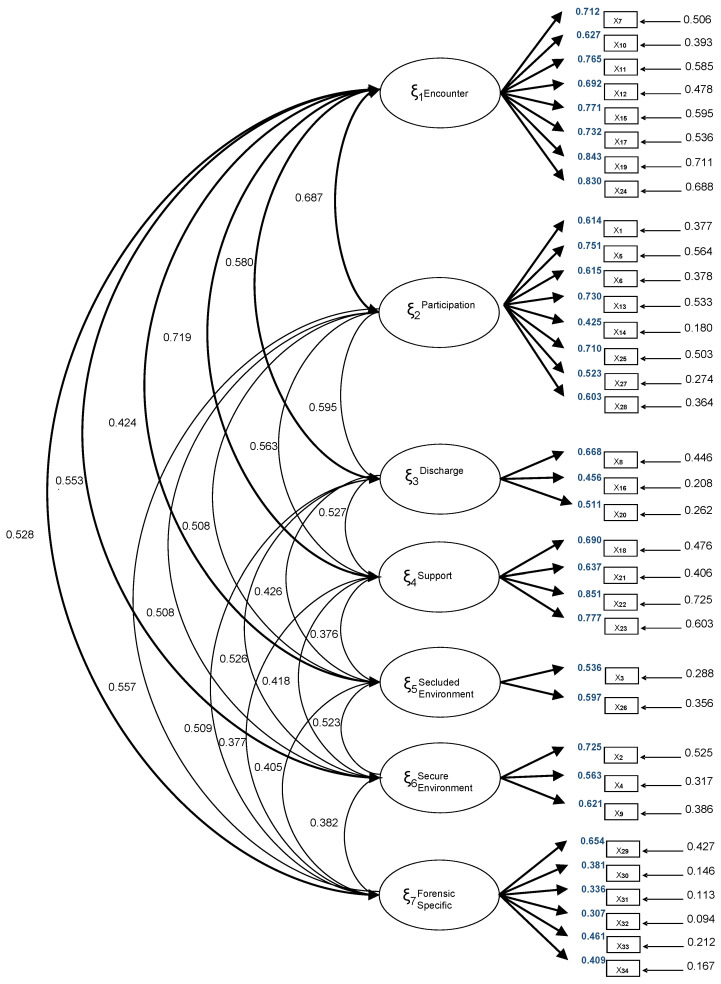
Factor loadings derived from the least squares estimation (least squares). Confirmatory factor analysis (λij).

To verify the internal structure of the questionnaire, a confirmatory factor analysis (CFA) was employed, proposing a seven-dimensional model identical to the structure of the original version. Table 2 presents the model fit indices, and Figure 1 displays the factor loadings. Not all indices demonstrated a reasonable fit, and the majority of item loadings were equal to or greater than 0.50. Correlations between the subscales are shown in Figure 1. Additionally, the correlations between each subscale and the global instrument were determined, obtaining subscale–global correlation values for F1. Encounter (0.882), F2. Participation (0.871), F3. Discharge (0.747), F4. Support (0.754), F5. Secluded Environment (0.619), F6. Secure Environment (0.682), and F7. Forensic-Specific (0.718).

### Reliability

The overall scale demonstrated high internal consistency with a Cronbach’s alpha coefficient of 0.933 and a McDonald’s omega coefficient of 0.940, indicating high overall reliability. However, some individual factors, specifically Factors 3, 5, and 7, presented a Cronbach’s alpha less than 0.70, but these factors presented a McDonald’s omega coefficient greater than 0.70 indicating a general high reliability.

The intraclass correlation coefficient (ICC) analysis demonstrated a test–retest reliability of 0.836 (95% CI: 0.742–0.896; *n* = 98), with this value being above 0.70 in all instrument factors except for Factor 3. Discharge (0.667, 95% CI: 0.476–0.788). The Cronbach’s alpha and ICC values are displayed in Table 3.

## 4. Discussion

The aim of this study was to perform a cross-cultural adaptation of the Quality in Psychiatric Care—Forensic Inpatient (QPC-FIP) instrument into Spanish and to assess its reliability and validity.

Regarding the adaptation of the instrument, translation–backtranslation procedure from the original version [28] did not reveal any significant problems. These findings are in line with those observed in the Danish adaptation of the instrument [37], and in this sense, it could be inferred that quality of care in forensic mental health setting has a similar essence to that attributed in the Swedish or Danish background.

When analyzing the results regarding psychometric properties, it can generally be stated that they were satisfactory, but some issues must be discussed. The confirmatory factor analysis (CFA) revealed a seven-factor structure equivalent to the original Swedish forensic version for patients [28] and staff [38], and no items required modification. These factor structures have also been observed in other adaptations of the forensic instruments, like the Spanish version of QPC-FIPS [27] and the Danish version of QPC-FIP [37] and QPC-FIPS [39]. The goodness of fit of the model it is reasonably improbable, but it is also justified by the low sample size, which can be explained by the low replacement rate that the forensic medium implies.

Regarding reliability, a global Cronbach’s alpha value of 0.933 was obtained, revealing values above 0.70 in most dimensions, which is considered a satisfactory result [36]. The total Cronbach’s alpha value is somewhat lower than that of the original Swedish instrument [28] and other versions of the QPC instrument [23,25,37,38,40,41,42], and very similar to the Danish forensic professional version [39]. It was, however, higher than the Spanish forensic professional, community, and hospital versions [22,24,27], Norwegian professional community version [43], Brazilian-Portuguese community patient version [44], and both Indonesian patient and professional versions [45,46]. Some factors showed a Cronbach’s alpha values below 0.70; specifically, this was the case for the factors F3. Discharge (α: 0.601), F5. Secluded Environment (α: 0.459), F6. Secure Environment (α: 0.672), and F7. Forensic-Specific (α: 0.569). Other versions of the QPC-FIP instrument (professional and patient versions) showed Cronbach’s alpha values below 0.70, and these common issues can be explained by the low number of items that compose each of the mentioned factors in F3. Discharge (three items) [27,37,38,39], F5. Secluded Environment (two items) [27,28,37,38,39], and F6. Secure Environment (three items) [27,37,38,39]. F7. Forensic-Specific showed the lowest value in comparison with the other forensic versions (both original and translated), and this issue can be explained by the small sample size of the study, being the lowest among all the forensic QPC versions [27,28,37,38,39]. With the intention of addressing this issue, McDonald’s omega was calculated to verify the internal consistency obtaining values over 0.70, which is considered adequate [36], in the previously mentioned factors F3. Discharge (ω: 0.795), F6. Secure Environment (ω: 0.775), and F7. Forensic-Specific (ω: 0.731), with the exception of F5. Secluded Environment (ω: 0.689), which was very close to the reference value.

Temporal stability was calculated as an additional indicator of reliability. This indicator was not analyzed in the original Swedish version [28], in most of the other original versions of the QPC instrument [38,40,42], or in their translated versions [37,39,44,45,46], except for the Spanish versions of the QPC instruments [22,23,24,25,27], the Norwegian professional community version [43], and the original professional hospital version [41]. The global ICC of the instrument and each of its factors is above 0.70, which in general it is considered a good agreement, except for the factor F3. Discharge (ICC: 0.667), which was close to the reference value. This instrument showed a global ICC somewhat lower than the Spanish hospital versions [22,23], Spanish professional community version [24], Norwegian professional community version [43], and original hospital version [41]. On the other hand, the instrument showed a higher ICC than the Spanish patients community version [25], and the Spanish forensic professional version [27].

Also, correlation values between the global instrument and each dimension were calculated, revealing that the correlation was higher between each dimension and the global instrument than correlations between dimensions, thus confirming the Fayer and Machin Hypothesis [47]. High correlations between the subscales and the overall score of the instrument suggest that these dimensions are not completely independent, which may complicate the interpretation of the results. However, this interrelation also highlights several strengths of the instrument, such as its ability to capture common aspects of the quality of psychiatric care, which reinforces its overall validity by reflecting a unified construct. In addition, the high correlations between the subscales indicate that the instrument may be sensitive to variations in the care provided, allowing the identification of general trends in service quality.

Below are the limitations to which this study has been subjected. The principal limitation arises from the small sample size of this study that may have an impact on the results as shown before. This limited sample size can be explained by the limited replacement rate linked to the forensic environment. Another limitation to highlight lies in the fact that this instrument was adapted to the forensic mental health context, and therefore, it cannot be used in other mental health contexts.

A relevant limitation of this study is the gender imbalance in the sample, which was predominantly composed of men. This bias could impact the psychometric analysis, as experiences and perceptions about the quality of care may differ between men and women. The lack of female representation limits the ability to generalize the results of the instrument to the entire forensic population, and could influence the stability of certain factors in the factor analysis, potentially affecting the validity of the instrument to capture the full experience of patients in forensic settings. As a strength, the study provides a valuable adaptation of the QPC-FIP instrument to the Spanish context, including the validation of its factor structure and internal consistency in a representative sample of male forensic patients, the predominant group in this type of units in Spain. This provides a robust and culturally adapted instrument, which may be useful for interventions targeting the majority population in forensic settings and establishes a solid foundation for future studies seeking to expand gender representativeness and improve the generalizability of the results.

A limitation is related to the impossibility of determining the sensitivity to change or predictive validity given the cross-sectional design of this study. Finally, the data necessary to allow the calculation of convergent validity were not collected. All these limitations should be taken into account in the design of future studies.

## 5. Conclusions

Below are a series of recommendations that are intended to serve as guidelines for developing future studies:

1. Expanding Samples and Contexts: One of the major limitations already mentioned in this study is the small sample size, which is justified by the low replacement rate. Future studies should work with larger samples. To improve the external validity of the instrument, it is recommended to carry out additional studies in other regions of Spain and in psychiatric contexts other than forensic, such as general hospitals or community care centers, additionally taking into account gender factors that guarantee an adequate female representation of the psychiatric population. This will allow researchers to check whether the identified quality factors are applicable in other environments and populations, favoring the generalization of the results and specific adaptations according to the needs of the context.
2. Longitudinal Evaluation of Sensitivity and Predictive Validity: It is essential to explore the sensitivity to change in the QPC-FIP instrument relating to the perception of quality over time and its ability to predict clinical and reintegration outcomes. Longitudinal studies that measure the perception of quality at different stages of the treatment process would allow researchers to evaluate the effectiveness of interventions in improving the quality of care, mental health status, and social reintegration in the long term.
3. International Comparative Studies: Given the variability in forensic care models between countries, it is suggested that international comparative studies be carried out to compare the results relating to quality of care in different mental health systems. This can contribute to the identification of good practices and international quality standards applicable to forensic psychiatric settings.
4. Inclusion of the Professionals’ Perspective: The integration of the perspective of mental health professionals in future studies could provide a more comprehensive view of the factors that affect the quality of care. The data collected from both points of view would allow the design of more balanced interventions and better coordination between care teams and patients.

The Spanish version of QPC-FIP is a helpful instrument for evaluating the quality of psychiatric care from the perspective of patients in a forensic context. Its seven-factor structure and psychometric properties are adequate and equivalent to its original version, allowing the measurement of quality of psychiatric care from the perspective of Spanish-speaking patients in the forensic context. A practical implication of using this instrument in clinical practice is that it allows for the continuous monitoring of the quality of care as perceived by patients in these settings, identifying specific elements that can be improved in response to the safety and well-being needs of patients in closed contexts.

Assessing the quality of forensic psychiatric care using the QPC-FIP instrument can guide the development of policies and specific interventions that promote empathetic therapeutic relationships, increase safety, and enhance patient engagement. This is critical in an environment where restrictive measures and the inherent control of the forensic context can limit patient empowerment and recovery. Furthermore, a higher quality of care in this context not only improves patients’ quality of life and well-being, but may also contribute to reducing recidivism, promoting more effective social reintegration.

## Figures and Tables

**Table 1:** Sociodemographic and clinical characteristics of the sample.

Background Variable	*n* (%)
**Gender**	
Female	15 (12.5%)
Male	105 (87.5%)
**Age M (SD)**	37.41 (10.91)
**Nationality**	
Spanish	83 (69.2%)
Others	37 (30.8%)
**Education level**	
Higher education/University	10 (8.3%)
Vocational training/Baccalaureate	39 (32.5%)
Primary education	42 (35%)
Incomplete education	29 (24.2%)
**Prior admission in forensic psychiatric unit**	
Yes	59 (49.2%)
No	61 (50.8%)
**Treatment**	
Pharmacological treatment	103 (85.8%)
Individual nursing treatment	63 (52.5%)
Group nursing treatment	38 (31.7%)
Psychological treatment	35 (29.2%)
**Treatment produced desired effect**	
Yes	98 (81.7%)
No	16 (13.3%)
**Perception after treatment**	
Worse	7 (5.8%)
Same	3 (2.5%)
Better	102 (85%)
Missing values	8 (6.7%)
**Knowledge of the diagnosis**	
Yes	69 (57.5%)
No	41 (34.2%)
Missing values	10 (8.3%)
**Diagnosis**	
Schizophrenia spectrum and other psychotic disorders	40 (33.3%)
Personality disorder	9 (7.5%)
Mood disorder	7 (5.8%)
Others	3 (2.6%)
Missing values	61 (50.8%)
**Current physical health**	
Good	69 (57.5%)
Neither good nor poor	26 (21.7%)
Poor	15 (12.5%)
Missing values	10 (8.3%)
**Current mental health**	
Good	79 (65.8%)
Neither good nor poor	24 (20%)
Poor	7 (5.9%)
Missing values	10 (8.3%)
**Participation in the planning of their care**	
Yes	89 (74.2%)
No	23 (19.2%)
Missing values	8 (6.7%)
**Readmission in the unit if necessary**	
Yes	62 (51.6%)
Not sure	16 (13.3%)
No	31 (25.8%)

**Table 2:** Goodness-of-fit indices of confirmatory model. Questionnaire: SPANISH-QPC-FIP.

Index	Value
BBNFI	0.597
BBNNFI	0.706
GFI	0.947
AGFI	0.938
CFI	0.734
RMR	0.074
SRMR	0.079
RMSEA	0.095
Cronbach’s α	0.933
Goodness-of-fit test	χ^2^ = 1048.436; df = 506; *p* < 0.0001
Reason for adjustment	χ^2^/df = 2.07

BBNFI: Bentler–Bonnet Normed Fit Index. BBNNFI: Bentler–Bonnet Non-Normed Fit Index. GFI: goodness-of-fit index. AGFI: adjusted goodness-of-fit index. CFI: comparative fit index. RMR: root mean square residual. SRMR: standardized root mean square. RMSEA: root mean standard error of approximation. Df: degree of freedom.

**Table 3:** Spanish QPC-FIP. Variables: ω; Cronbach’s alpha coefficient; test–retest ICC (*n* = 98).

Instrument factors	McDonald’s Ordinal Omega	Cronbach’s α	ICC (CI 95%)
Factor 1. Encounter (8 items)	0.950	0.907	0.854 (0.771–0.907)
Factor 2. Participation (8 items)	0.890	0.830	0.837 (0.745–0.896)
Factor 3. Discharge (3 items)	0.795	0.601	0.667 (0.476–0.788)
Factor 4. Support (4 items)	0.914	0.825	0.809 (0.701–0.879)
Factor 5. Secluded Environment (2 items)	0.689	0.459	0.708 (0.542–0.814)
Factor 6. Secure Environment (3 items)	0.775	0.672	0.889 (0.826–0.929)
Factor 7. Forensic-Specific (6 items)	0.731	0.569	0.777 (0.651–0.858)
Total	0.940	0.933	0.836 (0.742–0.896)

ICC, intraclass correlation coefficient; CI, confidence interval.

## Data Availability

The data presented in this study are available on request from the corresponding author due to ethical reasons.
